# Team-based learning for psychiatry residents: a mixed methods study

**DOI:** 10.1186/1472-6920-13-124

**Published:** 2013-09-11

**Authors:** Isabel McMullen, Jonathan Cartledge, Ruth Levine, Amy Iversen

**Affiliations:** 1 Liaison Psychiatry, South London and Maudsley NHS Foundation Trust, Guys Hospital, Weston Street, London SE1 9RT, UK; 2 Academic Centre for Medical Education, University College London, Gower Street, London WC1E 6BT, UK; 3 Department of Psychiatry and Behavioral Sciences, and Clinical Education, University of Texas Medical Branch, Galveston, TX 77555, USA; 4 Institute of Psychiatry, Weston Education Centre, Cutcombe Road, London SE5 9RJ, UK

**Keywords:** Instructional methods, Team-based learning, Continuing medical education, Residents, Psychiatry

## Abstract

**Background:**

Team-based learning (TBL) is an effective teaching method for medical students. It improves knowledge acquisition and has benefits regarding learner engagement and teamwork skills. In medical education it is predominately used with undergraduates but has potential benefits for training clinicians. The aims of this study were to examine the impact of TBL in a sample of psychiatrists in terms of classroom engagement, attitudes towards teamwork, learner views and experiences of TBL.

**Methods:**

Forty-four psychiatry residents participated in an Addictions Psychiatry TBL module. Mixed-methods were used for evaluation. Self-rated measures of classroom engagement (Classroom Engagement Survey, CES) were compared with conventional lectures, and attitudes regarding the value of teams (Value of Teams Scale, VTS) were compared before and after the module. Independent t-tests were used to compare ‘lecture’ CES scores with TBL CES scores and pre and post scores for the VTS. Feedback questionnaires were completed. Interviews were conducted with a subset of residents and transcripts analysed using thematic analysis.

**Results:**

Twenty-eight residents completed post-course measures (response rate 63.6%). Seven participants volunteered for qualitative interviews–one from each team. There was a significant difference in the mean CES score lectures compared to TBL (p < 0.001) but no difference was found in mean VTS score pre and post for either subscale (p = 0.519; p = 0.809). All items on the feedback questionnaire were positively rated except two regarding session preparation. The qualitative analysis generated seven themes under four domains: ‘Learning in teams’, ‘Impact on the individual learner’, ‘Relationship with the teacher’ and ‘Efficiency and effectiveness of the learning process’.

**Conclusions:**

In this group of residents, TBL significantly improved learner-rated classroom engagement and seemed to promote interactivity between learners. TBL was generally well-received, although required learners to prepare for class which was difficult for some. TBL did not change these clinicians’ views about teamwork.

## Background

Team-based learning (TBL) is an educational method suited to teaching the problem-solving and teamwork skills required in medicine [1-3]. In TBL, learners acquire basic knowledge by completing pre-session assignments and during sessions work together in small teams to apply knowledge to real-life situations. TBL enables active learning to be achieved with large classes.

For medical students, TBL has produced equal [4] or superior [5-9] academic outcomes compared to didactic teaching. The educational impact is thought to be greatest for students in the lowest academic quartile [10,11]. TBL improves student participation and engagement during class, as rated by learners [12], objective observers [13-15], and faculty [16,17]. TBL also promotes teamwork skills [7]. TBL has improved medical student satisfaction in the USA [7,9,17] and internationally [18-21]. Satisfaction increases as students and faculty become familiar with TBL: ratings increased each year as TBL became established [22,23].

While there is more limited experience of using TBL with clinicians, the data that exist suggest that TBL holds considerable promise for residency education. [12,24-26]. When compared to a lecture-based course for residents, TBL led to a similar increase in knowledge with greater participant engagement, although residents perceived TBL to have less educational value than lectures [14]. Using modified TBL to replace a lecture series led to improved knowledge scores with high ratings for perceived knowledge acquisition and enjoyment of interactive team discussions [26]. When primary care residents were given TBL “booster sessions” to learn the skill of alcohol screening and brief intervention, TBL was well received and reinforced their acquisition of a new clinical skill [24]. In a psychiatry residency training program focusing on the acquisition of psychotherapy skills, residents rated the presentation format as excellent, and specific comments about the TBL experience were overwhelmingly positive [25]. Since TBL moves beyond the basic acquisition of facts to focus on real life scenarios, it is an ideal didactic method for clinicians. As clinicians are used to solving patient problems and learning from them, TBL may be a natural way of learning for doctors in residency training.

The aims of this investigation were to determine whether, in a group of residents studying Addictions Psychiatry, TBL affected self-rated engagement and attitudes towards teamwork during a six-week teaching programme. We also aimed to assess doctors’ experiences of TBL.

## Methods

A mixed-methods observational study design was used. This was a pragmatic approach to evaluating the course. An alternative design such as a randomised controlled trial was rejected as it was not practical to run two parallel courses for the numbers of residents who were enrolled on the course.

### Setting

Participants were Core Psychiatry Trainees (residents) on the largest psychiatry training programme in the UK (http://maudsleytraining.com). We modified an existing weekly training course consisting of lectures grouped into curriculum-themed modules. The Addictions Psychiatry module was selected to pilot TBL. It consisted of 12 hours, divided into six two-hour sessions. A TBL expert visited to give a lecture and workshop to train faculty, review written materials and co-facilitate a session.

### Recruitment

All residents were sent information about the TBL module and evaluation prior to the start. There were no exclusion criteria: all residents attending the module were invited to participate in the evaluation. Sample size was limited by the numbers of residents attending the module, thus a sample size calculation was not undertaken.

### Structure of the module

The orientation session included a demonstration of TBL, based on a presentation from the TBL Collaborative website [27]. During the session, residents were grouped into learning teams of 6–7 residents using the ‘line-up’ method [3]; learners were lined up according to previous Addictions Psychiatry experience and numbered into teams. This ensured that teams had approximately equal distribution of members with expertise in Addictions Psychiatry.

The TBL sessions ran from May to June 2012. The sessions were led by different subject experts each week, and co-facilitated by the researcher with TBL training. Each session followed the same format. Preparatory material was emailed the week before. In each session, participants sat the Individual Readiness Assurance Test (IRAT): 8–10 multiple choice questions with no access to materials or peer discussion. Following the IRAT, teams worked on the same questions together (Group Readiness Assurance Test, GRAT), using IF-AT (Immediate Feedback Assessment Technique) scratch cards to gain immediate feedback. The GRAT scores were posted at the front of the room. The expert clarified questions arising from the RAT. Teams then worked through Application Activities, reaching consensus on answers though intra-team discussion. When prompted by facilitators, all teams simultaneously revealed their answer by holding up a card marked with a letter to indicate their choice. The facilitators led inter-team discussions about the Applications. For examples of the materials used, see the ‘Sample materials’ in Appendix section.

### Measures

#### *Learner-rated engagement*

The Classroom Engagement Survey (CES) [28] contains eight items measuring learner participation and enjoyment of class. It has been validated in a previous TBL study [8]. Items are scored on a 5-point Likert scale; maximum score range 5–40; higher scores indicate greater engagement. The questionnaire asks participants to rate the session that has just finished. For a comparison measurement, all participants who were present completed the CES immediately after a lecture from the previous module which was held just before the start of the preliminary TBL session. This was a typical lecture which was the standard method of teaching prior to the introduction of TBL. For the active measurement, participants completed it immediately after the final TBL session. This enabled comparison of engagement during a typical TBL session with that found during a typical lecture.

#### *Attitudes towards teamwork*

The Value of Teams survey (VTS) [29] measures 17 items in two dimensions: ‘value of group work’ and ‘working with peers’. Items are scored on a 5-point Likert scale. Higher scores indicate perceptions of greater value of learning in teams (subscale scores range 6–30). The VTS was carried out at the same time points as the CES.

#### *Feedback questionnaires*

We created a 20-item questionnaire based on one used in previous TBL evaluations [30]. This was completed after the final TBL session. For each item, respondents used a 5-point Likert scale, scored from −2 to +2. There were 20 items in total, with eight items making up each of the two factors: perceptions of teamwork and perceptions of TBL (factor scores ranged −16 to +16). Positive scores indicated favourable perceptions. The free text section of the questionnaire asked:

1. What did you like about TBL?

2. What did you not like about TBL?

3. Any other comments about TBL?

### Statistical analysis

CES and VTS scores for each participant were entered on an Excel spreadsheet. SPSS was used to compare the variances of the samples and to calculate the difference in scores after the lecture and after the TBL session using a t-test. An independent t-test was used rather than a paired test as it could not be assumed that the same participants completed the pre and post questionnaires, due to varying participant attendance at the first and final sessions. As the questionnaires were completed anonymously, it could not be guaranteed that the samples were paired.

### Qualitative interviews

Semi-structured interviews lasting 45 minutes to 1 hour were conducted with a volunteer from each team to enable the broadest sampling of views. Interviews were conducted by one researcher and were audio-recorded, then transcribed. Transcripts were stored in accordance with the Data Protection Act 1998. Transcripts were reviewed by the interviewee for accuracy and analysed using thematic analysis, which was chosen to allow for theoretical freedom during analysis.

### Ethical considerations

Guidance was sought from the Psychiatry, Nursing and Midwifery Ethics Committee at King’s College London, University College London Ethics Committee, and the South London and Maudsley NHS Foundation Trust/Institute of Psychiatry Research and Development Office. All offices agreed in writing that the project was an evaluation project rather than research and thus did not require full ethical approval. Information sheets were given to all participants who gave written consent to participate.

## Results

All 44 residents who attended the module participated in the evaluation. 22 (50%) were female, 22 (50%) were male. Three (7%) had previous experience in Addictions Psychiatry. 26 residents completed initial measures; 28 completed post-course measures (response rate 63.6%); 7/44 (16%) were interviewed, one from each team.

### CES

An independent t-test was used to compare the difference between the mean CES score following the didactic session and the CES score following the TBL module. An unequal variance t-test was used as the samples had different variances. A sensitivity analysis showed one outlier in the post group who was excluded and the t-test was run again, with no great difference in outcome. The mean CES score following the conventional didactic lecture was 25.5, the CES following the TBL session was 32.3, mean difference 6.8 (CI 3.4–10.1), p < 0.001.

### VTS

Independent t-tests were used to compare pre and post scores on both subscales of the VTS: Value of Peers and Value of Group Work. For both subscales, the samples had equal variances and so an equal variance t-test was used. No significant difference was found in either subscale between the pre and post samples: Mean Value of Peers score pre = 24.8, post = 24.3, mean difference 0.45 (CI 0.93–1.83), p = 0.519; Mean Value of GroupWork score pre = 24.11, post = 24.28, mean difference 0.16, (CI 1.19–1.52), p = 0.809.

### Feedback questionnaire

Item scores ranged from −0.72 to +1.52 (Table 1). Eighteen (out of 20) items were positively scored, indicating an overall positive perception of TBL. The highest scoring items were: ‘the ability to work in a team is necessary if I am to be successful as a psychiatrist’ and ‘I have a positive attitude about working with others in a team’. The two negatively scored items were: ‘I found it easy to complete the pre-session reading’ and ‘I generally felt prepared for the IRAT’.

**Table 1:** Feedback questionnaire results

**ITEM**	**MEAN**
**1. TBL helped me increase my knowledge of addictions psychiatry**	1.03
2. I found it easy to complete the pre-session reading	-0.72
**3. I found the pre-session reading helpful**	0.28
**4. Individual readiness assurance tests (IRAT) were useful learning activities**	0.59
5. I generally felt prepared for the IRAT	-0.10
**6. The GRAT (group) discussions allowed me to improve my understanding of concepts**	0.76
7. The GRAT (group) discussions were useful learning activities	0.97
8. I learn better from TBL than from lectures	0.45
**9. Solving problems in a group is an effective way to learn**	0.83
**10. I learned useful additional information during the TBL sessions**	1.03
**11. TBL helped me prepare for MRCPsych examinations**	0.03
*12. I have a positive attitude about working with others in a team*	1.24
*13. The ability to work in a team is necessary if I am to be successful as a psychiatrist*	1.52
*14. Solving problems in a group is an effective way to practice what I have learned*	1.14
*15. My team worked well together*	1.00
*16. I contributed meaningfully to the TBL discussions*	0.97
*17. Most students were attentive during TBL sessions*	0.86
*18. I paid attention most of the time during the TBL sessions*	0.86
**19. The TBL format was helpful in developing my skills for clinical practice**	0.59
*20. There was mutual respect for other teammates’ viewpoints during TBL*	1.10
**Total score for ‘perception of TBL’ factor**	**5.14**
***Total score for ‘perception of teamwork’ factor***	**7.76**

‘Perception of TBL’ items are in **bold** and ‘perception of teamwork’ items are in *italics*.

### Themes from interviews

Thematic analysis revealed seven themes that were grouped under four domains:

#### *Learning in teams*

**Learning knowledge in teams** Interaction between team members consolidated previously learnt knowledge and enabled learning of new knowledge. The discussions led to deeper understanding and better retention:

‘You’re not just learning the answers, you’re problem-solving, formulating arguments to justify your position…the reasoning…means deeper learning…’ [P2]

Individuals were motivated to study and concentrate as they felt responsible for their team’s performance.

**Learning about group dynamics** The TBL process meant that teamwork skills were developed: communication skills, self-awareness, leadership, negotiation techniques, and respect for others. Teams actively managed interactions or let them naturally evolve. Teams became more cohesive during the module:

‘We didn’t know each other…now we’re much more…interactive, challenging each other. It’s great’ [P5]

**Social aspect of TBL** Learning was ‘fun’, ‘enjoyable’. This was enhanced by scratch-cards and competitive element:

‘I love all that competitiveness. I really want to get the top score, win the prize’ [P4]

TBL promoted interaction between different friendship and cultural groups:

‘I would never have spoken to her if it hadn’t been for us being in the same group’ [P3]

Being part of a team meant that individuals were less fearful of answering questions in front of the class.

#### *Impact on the individual learner*

**Impact on individuals’ knowledge acquisition** The preparatory reading structured learners’ personal study, and the deadline was motivating for some. However there were differing views about whether the preparation was excessive. Participants found that completing the reading made the session more useful.

‘It reactivates stuff I…know already…that makes that knowledge stick…more…you understand better, and then you remember it longer…’ [P5]

The IRAT acted as a form of self-evaluation and was useful for individuals to assess how much they had learnt by preparing. The Applications were relevant to residents’ clinical practice and were thought to improve clinical skills:

‘The focus…on using the info you’ve learnt rather than just acquiring it… you come out … a better doctor… it’s so much more useful [than lectures]’ [P6]

Applications encouraged reflection on clinical work which increased the material’s relevance.

**Impact on learning other skills/general qualities of TBL** TBL promoted punctuality and engagement:

‘because you’re never doing the same thing for more than 15 minutes…, keeps your attention… impossible not to be…engaged in what you’re doing… no-one’s on their phones or email…so different’ [P7]

Participants felt that the interactivity was more ‘natural’ than in lectures. It enabled learners to feel comfortable addressing the whole class.

#### *Relationship with the teacher*

The increased interactivity led to a less formal relationship in which it was easier to ask questions:

‘it feels natural to ask questions…totally different to a lecture when it disturbs the flow if you ask a question’ [P2]

The RAT meant that teachers were aware of learners’ baseline knowledge, which meant the session was pitched at correctly. Applications promoted discussion, enabled teachers to use clinical examples, and helped residents improve clinical decision-making skills.

#### *Efficiency and effectiveness of the learning process*

Participants viewed lectures as an efficient method of delivering a large amount of information and expressed concerns about whether TBL took longer to cover less material:

‘I’m not sure we’re covering as much as we do in lectures… it’s going in more, but it takes a long time to cover a few… concepts’ [P3]

Learners found discussions helpful but were concerned they sometimes got ‘distracted’ by details. TBL was thought to be excellent at covering basic concepts, but learners felt that it was less effective at conveying advanced material and did not permit experts to present their latest research.

## Discussion

These findings show that for this group of psychiatry residents, TBL led to a significant improvement in classroom engagement compared to learning via conventional lectures. However residents demonstrated no change in attitudes regarding the value of teams as measured by the VTS.

Does classroom engagement matter? A meta-analysis of the effectiveness of Continuing Medical Education (CME) found that lectures had no impact on clinician performance or patient outcomes, even though they may increase knowledge short-term [31]. However, interactive teaching had a small effect on professional performance and on occasion, healthcare outcomes, i.e. a positive impact on patient care. It has been suggested that physicians get most from CME if their learning is self-directed and derived from true-life clinical settings [32]–difficult to achieve in a lecture-based programme, but more realistic with TBL. Further, evidence suggests that bored learners learn less deeply [33], and that learners in a positive mood have improved recall [34] and working memory for words [35]. If we want learners to learn and retain more, increasing engagement will help.

Participants’ ratings of the value of teams did not change during the module. This was unexpected as student studies showed that TBL caused improvement in individuals’ appreciation of teamwork. Potentially this group of doctors were more aware of the advantages of learning in teams as a result of working in teams, as compared to students. For junior psychiatrists who work particularly closely with multidisciplinary teams [36], appreciation for the value of teamwork is especially relevant.

The qualitative data and questionnaire results show that participants held generally positive views about TBL. However there were two items with a mean negative score, both regarding pre-class preparation. Participants reported difficulty finding time to prepare because of conflicting demands from professional or personal commitments. Similar problems have been reported by others using TBL for residents [37]. These researchers asked residents to complete the preparatory reading and IRAT before class. Although attendance, satisfaction, and knowledge outcomes were good, only a third of residents completed the IRAT, suggesting that excluding it from class-time was not successful.

A challenge remains for those using TBL with residents: incentivizing residents to complete preparatory work may be more difficult than incentivizing students, who are largely motivated by grades. It is possible that our readings were too long, and may have been more acceptable to the residents if we had been more judicious when choosing pre-class preparation. Alternatively, we might have increased incentives for completing it if we had increased the rewards for doing well on IRATs and GRATs.

It may be useful for clinical teachers to know what aspects of TBL were thought to be the most effective, so that they may use these elements, even if they do not have time to carry out a complete TBL session. From the qualitative results, residents stated that the opportunity to work through real-life clinical scenarios with peers and an expert was the most beneficial aspect of TBL.

An important research question is to discover whether TBL produces more clinically competent residents than those trained by traditional courses. These results indicate that residents felt that TBL helped clinically, but this does not necessarily translate into improved performance.

### Limitations

The lack of exclusion criteria meant that the sample was as broad and representative as possible of this population. Nonetheless, participants were recruited from one year of one training programme in psychiatry and therefore the results may have limited generalizability. The selection of one module meant that residents only received TBL for one topic area, from one group of faculty. Views held about the topic may have influenced views about TBL. Repeating the measures when TBL is used to teach other modules would enable comparison to ensure that attitudes were not topic-specific.

The measures were self-rated and subjective. An objective observation tool [15] was considered for measuring classroom engagement, but was rejected due to resource implications and concern that observers might disrupt sessions. The CES comparison also asked participants to compare a typical lecture with a typical TBL session. It may have been that the two sessions selected were not typical of lectures or TBL.

## Conclusions

TBL led to improvement in learner-rated classroom engagement in this sample of residents. Greater classroom engagement may lead to greater learning and interactivity, which has been shown to improved clinical outcomes for patients. This study also shows that TBL was generally well-received, although required learners to prepare for class, something which was difficult for our busy clinicians to accept. In this sample, residents’ views about teamwork were not altered by TBL which was different than the effect on students, however this may be because of a pre-existing high levels of appreciation of the value of teams in this sample.

## Appendix

### Sample materials

#### *Example of a RAT question*

Which of the following is an example of classical conditioning with regards addiction behaviour?

a. A heroin-dependent man develops heroin cravings when he sees a needle, spoon and syringe*

b. A group of friends who take heroin together socially and have done so from a young age

c. A heroin-dependent man who uses heroin because it acts to alleviate the symptoms of withdrawal

d. A heroin-dependent man who uses heroin because it causes a euphoric state

e. A man who uses heroin to relax because his father and brothers do so

#### *Example of an application activity*

A 24 year old man is remanded to prison. On assessment he reports injecting 1 g of heroin a day, smoking 2 rocks of crack cocaine a day, taking 20 mg of illicit diazepam a day and drinking 6 cans of 9% beer every day. He is requesting opioid substitution treatment (OST) and an alcohol detoxification.

1.1  What single piece of information from the history would be most useful to help you decide whether to prescribe OST or not?

A) Time/amount of last drug use

B) Whether he was on OST in the community

C) Known drug allergies

D) Whether he currently feels he is suffering from opioid withdrawal symptoms

E) Past medical history

He tells you he last used over 24 hours ago and reports significant subjective opioid withdrawal symptoms. You carry out a physical examination.

1.2  Which of the following sets of findings would make you most likely to prescribe OST?

A) Tremor, sweating, dilated pupils, track marks

B) Drowsiness, heart rate 64 bpm, constricted pupils, track marks

C) Hypertension, agitation, sweating, tremor

D) Low weight, sweating, heart rate 72 bpm, tremor

E) Dilated pupils, heart rate 92 bpm, hypertension, sweating

You determine that he is in opioid withdrawal and you decide to prescribe OST. What would be the most appropriate investigation to support your decision?

A) Blood alcohol concentration

B) Urine drug screen

C) Hair strand testing

D) Saliva swab test

E) Blood screen (including LFTs)

Considering his alcohol use, he tells you that he drinks six cans of 9% abv beer every day. He reports that this has increased over the past six months since he was last in prison, but he no longer feels intoxicated after drinking. He is unaware of any health problems as a result of his alcohol consumption and denies “needing” to have a drink, or any physiological symptoms of alcohol withdrawal.

1.3  What is the best management of his alcohol use?

A) 5-day chlordiazepoxide reducing regime (starting at 20 mg qds)

B) Monitoring of observations but no medication

C) Chlordiazepoxide prn

D) No treatment required

E) 3-day chlordiazopoxide reducing regime (starting at 10 mg qds)

You decide to commence a chlordiazepoxide reducing regime and prescribe 20 mg qds. He is also started on methadone 30 mg and given a dose. The following morning you are asked to review the man. He is drowsy but rousable, with constricted pupils, and a heart rate of 58 bpm.

1.4  What is your best immediate course of action?

A) Stop the chlordiazepoxide

B) Administer naloxone intramuscularly

C) Move to medical wing for physical observation monitoring

D) Omit the next dose of methadone

E) Reduce the next dose of methadone to 20 mg

## Abbreviations

TBL: Team-based learning; CES: Classroom engagement scale; VTS: Value of teams survey; CT: Core trainee (in psychiatry); CME: Continuing medical education.

## Competing interests

The authors report no competing interests.

## Authors’ contributions

IM and AI conceived of the module and of the study. IM, AI and RL reviewed the materials for the module. IM, JC and AI designed the study. IM collected and analysed the data, and drafted the manuscript. All authors reviewed and approved the final manuscript.

## Authors’ information

Isabel McMullen is a Specialist Registrar in Liaison Psychiatry at Guys Hospital, South London and Maudsley NHS Foundation Trust.

Jonathan Cartledge is the director of the Royal College of Physicians/ University College London MSc, diploma and postgraduate certificate in Medical Education. He is a senior lecturer in Medical Education at UCL, and a consultant HIV physician at the Mortimer Market Centre.

Ruth E. Levine is the Clarence Ross Miller Professor of Psychiatry for the Department of Psychiatry and Behavioral Sciences and the Assistant Dean of Clinical Education at the University of Texas Medical Branch in Galveston, Texas.

Amy Iversen is a Consultant Liaison Psychiatrist and a Senior Lecturer in Medical Education at the Institute of Psychiatry, UK.

## Pre-publication history

The pre-publication history for this paper can be accessed here:

http://www.biomedcentral.com/1472-6920/13/124/prepub
